# Effectiveness of bone grafting versus cannulated screw fixation in the treatment of posterolateral tibial plateau compression fractures with concomitant ACL injury: a comparative study

**DOI:** 10.1186/s13018-023-04516-8

**Published:** 2024-01-17

**Authors:** Yang Yang, Xiaofang Lin, Jianmin Zhang, Hanlong Xin, Dawei Han, Qingguo Zhang, Xiaobo Zhou

**Affiliations:** Department of Orthopedics, Taizhou Hospital of Zhejiang Province, Affiliated to Wenzhou Medical University, No. 150 Ximen Street, Linhai City, 317000 Zhejiang Province China

## Abstract

**Background:**

Posterolateral tibial plateau compression fractures (PTPCF) are one of the significant factors leading to knee instability and anterior cruciate ligament (ACL) reconstruction failure. The effectiveness of fixation for such cases without the use of metal implants remains inconclusive. The aim of this study is to investigate whether the fixation with isolated bone grafting is stable enough for the treatment of PTPCF with concomitant ACL injuries.

**Methods:**

This retrospective study analyzed patients treated for concomitant ACL injuries and PTPCF in authors’ institution. A total of 53 patients (21 males and 32 females) with an average age of 47.43 ± 14.71 years were included. Patient data were collected, including factors leading to injury, affected side, height, weight, and basic medical history. The posterior inclination angle and the lateral tibial plateau lateral inclination angle were measured to evaluate the fixation stability. Rasmussen functional score and HSS score were used to assess the knee functional recovery.

**Results:**

The bone grafting group achieved satisfactory levels of Rasmussen score (28.22 ± 0.85) and HSS knee joint function scores (95.57 ± 1.97). The cannulated screw fixation group had a Rasmussen knee joint function score of 28.70 ± 0.92 and a HSS knee joint function score of 96.07 ± 1.93. No statistically significant difference was found (*P* > 0.05). The cannulated screw fixation group had a mean posterior inclination angle reduction loss of 0.20° ± 1.11°, while the bone grafting group had a reduction loss of 0.18° ± 1.01°, with no statistically significant difference (*P* > 0.05). The cannulated screw fixation group had a lateral inclination angle reduction loss of 0.01° ± 0.37°, and the bone grafting group had a reduction loss of 0.03° ± 0.43°, with no statistically significant difference (*P* > 0.05).

**Conclusion:**

The use of bone grafting for fixation of PTPCF with accompanying ACL injuries demonstrated no substantial disparities in knee joint function. In cases of simple PTPCF, filling and compacting the bone defect underneath the tibial plateau fracture fragment can yield satisfactory fixation, obviating the necessity for supplementary cannulate screw fixation.

## Introduction

Posterolateral tibial plateau compression fractures (PTPCF), accounting for only 7–15% of all tibial plateau fractures, are closely associated with anterior cruciate ligament (ACL) injuries or ACL attachment avulsion fractures [1–4]. The posterolateral tibial plateau plays a crucial role in knee flexion stability, and fractures in this region are often accompanied by an increased posterior inclination angle or an increased lateral inclination angle of the lateral plateau [5, 6]. An increased posterior inclination angle or lateral inclination angle of the lateral plateau can lead to increased load on the ACL during knee joint motion, closely related to ACL injuries, and is one of the significant factors leading to knee instability and ACL reconstruction failure after ACL reconstruction surgery [7]. Therefore, to achieve satisfactory clinical outcomes and reduce the failure rate of ACL reconstruction, the treatment of PTPCF with concomitant ACL injury should not be limited to ACL reconstruction alone; equal attention should be given to the treatment of PTPCF [8, 9]. While addressing PTPCF in patients with concomitant ACL injuries, some surgeons use arthroscopic-assisted reduction and cannulated screw fixation [10]. Other orthopedists perform arthroscopic-assisted reduction and fixation with bone grafting only [11]. Nevertheless, the effectiveness of fixation for these fracture types without the use of metal implants is yet to be conclusively established. Therefore, this study retrospectively analyzed patients who had concomitant ACL injuries and PTPCF to investigate whether the fixation with isolated bone grafting is stable enough for the treatment of PTPCF with concomitant ACL injuries. The hypothesis of this study was that fixation with isolated bone grafting for PTPCF with concomitant ACL injuries can get a stable fixation as well as satisfactory clinical and radiological outcomes compared to fixation with cannulated screws.

## Materials and methods

This retrospective study analyzed patients treated for concomitant ACL injuries and PTPCF in authors’ institution between June 1, 2017, and May 31, 2022.

Inclusion criteria: (1) preoperative knee joint magnetic resonance imaging (MRI) showing ACL injury or ACL attachment avulsion fractures, with three-dimensional computed tomographs (3DCT) confirming PTPCF; (2) articular step-off of posterolateral tibial plateau fractures ≥ 2 mm or lateral tibial plateau posterior inclination angle greater than or equal to 17°, with patients undergoing arthroscopic surgery for tibial plateau fracture reduction; (3) age ≥ 18 years; (4) follow-up duration ≥ 12 months; and (5) complete preoperative and postoperative imaging data.

Exclusion criteria: (1) intraoperative confirmation of no ACL injury; (2) patients undergoing isolated ACL reconstruction or ACL avulsion fracture reduction and fixation; (3) multiple injury patients; (4) cases of open fractures; (5) patients with severe osteoarthritis; (6) age < 18 years; (7) follow-up duration less than 12 months; and (8) patients treated conservatively.

This study included a total of 53 patients (21 males and 32 females) with an average age of 47.43 ± 14.71 years (range 18–72 years). The patients were diagnosed with concomitant ACL injuries and PTPCF with ACL reconstruction and tibial plateau fracture reduction and fixation as a one-stage procedure. Patients were divided into two groups based on whether the PTPCF was internally fixed with cannulated screws during the procedure: the cannulated screw fixation group (30 cases, 13 males, and 17 females) and the bone grafting fixation group (23 cases, 8 males, and 15 females) (Table 1). This study was approved by the Medical Ethics Committee of Taizhou Hospital in Zhejiang Province, and all enrolled patients were informed and signed informed consent.

**Table 1:** Baseline data comparison between the cannulated screw fixation group and the isolated bone grafting fixation group

Variable	Cannulated screw fixation group	Bone grafting fixation group	*P* value
Numbers	30	23	–
Sex (male/female)	13/17	8/15	0.528
Age (years)	46.60 ± 15.84	48.52 ± 13.36	0.642
BMI	24.45 ± 2.34	24.54 ± 1.90	0.880
Injury side (left/right)	14/16	10/13	0.817
Hypertension (*n*)	4	5	0.478
Diabetes (*n*)	4	2	0.687
Injury causes			0.649
Traffic accident (*n*)	23	15	
Sports injury (*n*)	5	6	
Fall (*n*)	2	2	
Medial collateral ligament injury (*n*)	17	12	0.745
Meniscus injury (*n*)	22	17	0.962
Thrombosis (*n*)	4	3	1.000
Type of plateau inclination			0.450
Posterior inclination (*n*)	20	13	
Lateral inclination (*n*)	10	10	
ACL reconstruction (*n*)	22	21	0.158

*BMI* body mass index, *ACL* anterior cruciate ligament

### Surgical procedure

Patients were placed in the supine position, and after the onset of general anesthesia, anterior and posterior drawer tests, Lachman tests, varus/valgus stress tests, and knee joint flexion–extension assessments were performed to evaluate knee joint mobility and stability. Knee joints were flexed to 90 degrees, and medial and lateral approaches were established. After removing intra-articular hematomas and synovial debris to ensure a clear surgical field, intra-articular injuries were examined, including articular fracture fragments, articular surface injuries, ligament ruptures, and meniscus tears.

**Fig. 1:**
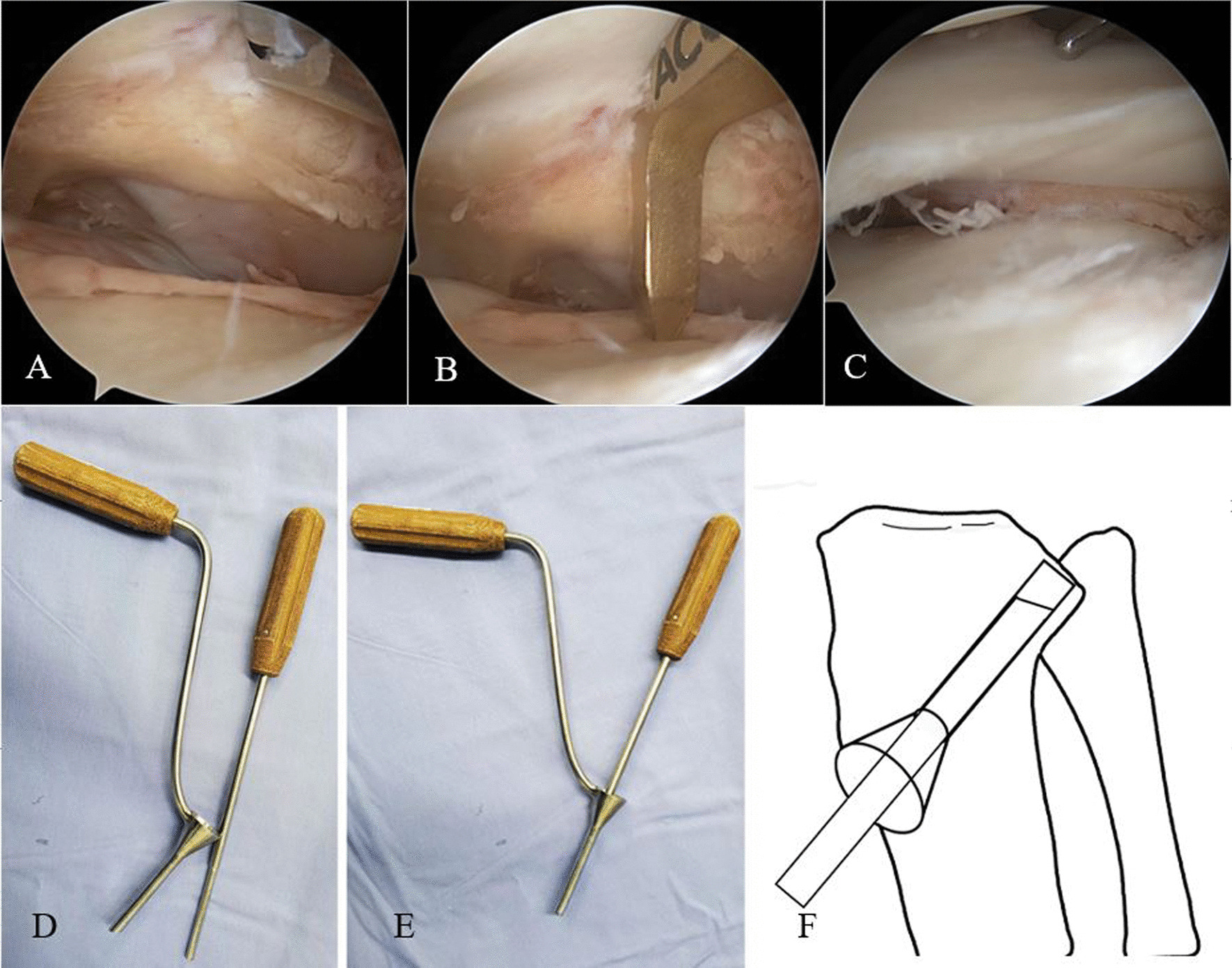
Following the clearance of synovium and blood within the joint cavity, the posterior aspect of the lateral meniscus is lifted to expose the tibial plateau fracture (**A**). Utilizing an anterior cruciate ligament guide, the articular surface of the fracture is localized at a lower position (**B**), and a Kirschner wire is used to establish a bone tunnel. A metal tamp is used to gently tap the depressed articular surface, thereby restoring the height and angular orientation of the depressed posterior lateral articular surface (**C**). A metal tamp was used to elevate the fracture fragments through the prefabricate 7.0-mm cannula, and a bone grafting funnel was used to fill the defect under the tibial fracture fragments with allograft bone or calcium sulfate artificial bone (**D**–**F**)

Aiming to locate the lowest point of the articular fragment, the ACL guide was used to insert a 2.0-mm Kirschner wire from anterolateral portal, creating a 7.0-mm tunnel, which was then used to elevate the collapsed articular surface with a metal tamp (Fig. 1A–C) [12]. The reduction of articular surface was confirmed using arthroscopy. Then inserting a bone graft funnel into the fabricated 7.0-mm tunnel and filling and compacting the bone defect under the tibial plateau fracture fragments with allograft bone (Osteolink, China) or calcium sulfate artificial bone (Biocomposites, UK) for the bone grafting group (Fig. 1D–F). In the cannulated screw fixation group, temporary fixation was carried out with the 2.0-mm Kirschner wires from the lateral plateau to the medial plateau when deemed necessary after reducing the fracture. One to two 7.3-mm cannulated screws (Canwell, China) were implanted below the fracture fragments to get a rigid fracture fixation without bone grafting.

Meniscus reshaping or repair surgery was performed when needed. For medial collateral ligament ruptures, traditional repair methods were used. In cases of ACL attachment avulsion fractures, a 2-0 Ethibond suture was used for traction reduction and fixation. Autogenous hamstring tendons or gracilis tendons were used as grafts for single-bundle ACL reconstruction [10].

### Postoperative recovery and follow-up

All patients underwent standardized postoperative recovery and guidance training. Starting from the first day after surgery, quadriceps muscle strength training began, with passive knee flexion exercises at 30 degrees of flexion. Two weeks after surgery, patients began non-weight-bearing ambulation with knee joint support. After four weeks, partial weight-bearing ambulation commenced, and at eight weeks post-surgery, full weight-bearing ambulation was allowed. All enrolled patients underwent a minimum of 12 months of follow-up, during which their knee joint mobility, HSS knee joint function score (Hospital for Special Surgery), Rasmussen knee joint function score, and VAS (visual analog scale) for pain assessment were conducted.

### Clinical and radiological assessment

Patient data were collected, including factors leading to injury, affected side, body mass index (BMI), and basic medical history such as hypertension and diabetes. Radiological data, comprising preoperative and postoperative knee joint lateral images, 3DCT scans, and knee joint MRIs, were collected to evaluate the patient's condition before and after the operation. Preoperatively, lower limb vascular Doppler ultrasound examinations were routinely performed to rule out deep vein thrombosis. If no deep vein thrombosis was detected, low-molecular-weight heparin calcium injection (4100 IU) was administered subcutaneously to prevent lower limb deep vein thrombosis. If deep vein thrombosis was present, treatment with low-molecular-weight heparin calcium injection (4100 IU) every 12 h subcutaneously was initiated. Postoperatively, lower limb sensation was assessed to evaluate potential peroneal nerve damage. Follow-up occurred over a minimum of 12 months and involved assessing knee joint mobility (extension and flexion), knee joint VAS pain scores, Rasmussen function scores, and HSS knee joint scores to evaluate postoperative knee joint function.

**Fig. 2:**
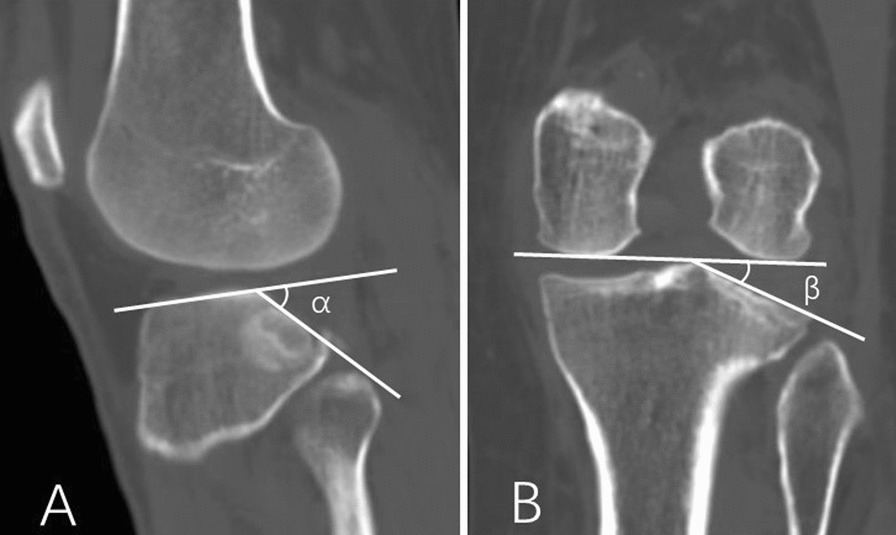
As illustrated in **A**, the angle α is formed between the tangent line of the lateral plateau and the tangent line of the collapsed posterior lateral articular surface, representing the lateral tibial plateau's relative posterior inclination angle. In **B**, the angle β is formed between the tangent line connecting the lowest edges of the medial and lateral femoral condyles and the tangent line of the collapsed lateral plateau articular surface, indicating the lateral plateau inclination angle

The PACS (picture archiving and communication system) was used to measure the radiological parameters of the tibia plateau, including the relative posterior inclination angle and the lateral tibial plateau lateral inclination angle before surgery, postoperatively, and at the final follow-up, in order to assess the alignment of the lateral tibial plateau (Fig. 2).

### Statistical

For statistical analysis, SPSS version 26.0 (SPSS Inc., Chicago, IL, USA) was used. Continuous variables were expressed as mean ± standard deviation. Independent-sample* t* tests were utilized for between-group comparisons. Categorical variables were compared using Chi-square tests or Fisher's exact tests to assess differences between the two groups. A significance level of *P* < 0.05 was considered statistically difference.

## Results

Baseline data, including patient age, gender composition, BMI, operated side, diabetes, hypertension, and follow-up time, did not differ significantly between the two groups (*P* > 0.05) (Table 1). Peroneal nerve injury was not observed in either group, and no cases of ACL reconstruction failure were noted. There were no significant differences between the two groups regarding knee joint periarticular soft tissue injuries (meniscus injuries, medial collateral ligament injuries), major tibial plateau inclination types, or incidence of deep venous thrombosis of lower extremity (*P* > 0.05) (Table 1).

At the final follow-up, the mean extension angle of the knee was 0.65° ± 1.72° (range 0°–5°) in the bone grafting group, with a mean flexion angle of 124.78° ± 7.46° (range 110°–140°). In the bone cannulated screw fixation group, patients had a mean extension angle of 0.50° ± 1.53° (range 0°–5°) and a mean flexion angle of 127.50° ± 8.78° (range 115°–145°). There was no statistically significant difference in knee joint mobility between the two groups (*P* > 0.05).

The isolated bone grafting group achieved satisfactory levels of Rasmussen score (28.22 ± 0.85) and HSS knee joint function scores (95.57 ± 1.97). Similarly, the cannulated screw fixation group had a Rasmussen knee joint function score of 28.70 ± 0.92, and a HSS knee joint function score of 96.07 ± 1.93. There was no statistically significant difference in knee joint function scores between the two groups (*P* > 0.05) (Table 2).

**Table 2:** Clinical outcome comparison between two groups

Variable	Cannulated screw fixation group (*n* = 30)	Bone grafting fixation group (*n* = 23)	*P* value
Follow-up (month)	15.10 ± 2.67	14.74 ± 3.37	0.665
VAS	0.30 ± 5.35	0.57 ± 0.66	0.123
Range of motion			
Extension (°)	0.50 ± 1.53	0.65 ± 1.72	0.735
Flexion (°)	127.50 ± 8.78	124.78 ± 7.46	0.240
Rasmussen functional score	28.70 ± 0.92	28.22 ± 0.85	0.055
HSS score	96.07 ± 1.93	95.57 ± 1.97	0.357

*VAS* visual analogue scale, *ROM* range of motion, *HSS* Hospital for Special Surgery

To further assess the reliability of maintaining articular surface reduction after bone grafting fixation, a comparison of posterior inclination angle and lateral inclination angle of the lateral tibial plateau was performed for patients before surgery, postoperatively, and at the final follow-up. It was found that there was no statistically significant difference in the posterior inclination angle and lateral inclination angle of the lateral tibial plateau at both the postoperative and final follow-up time points.

The cannulated screw fixation group had a mean posterior inclination angle reduction loss of 0.20° ± 1.11°, while the bone grafting group had a reduction loss of 0.18° ± 1.01°, with no statistically significant difference between the groups (*P* > 0.05). The cannulated screw fixation group had a lateral inclination angle reduction loss of 0.01° ± 0.37°, and the bone grafting group had a reduction loss of 0.03° ± 0.43°, with no statistically significant difference between the groups (*P* > 0.05) (Table 3).

**Table 3:** Radiological outcome comparison between two groups

Variable	Cannulated screw fixation group (*n* = 30)	Bone grafting fixation group (*n* = 23)	*P* value
Lateral tibial plateau posterior inclination			
Pre-operation	31.94 ± 7.09	30.22 ± 6.98	0.385
Post-operation	14.39 ± 1.65	15.02 ± 1.39	0.148
Final follow-up	14.60 ± 1.33	15.20 ± 1.14	0.086
Reduction loss (°)	0.20 ± 1.11	0.18 ± 1.01	0.945
Lateral tibial plateau lateral inclination			
Pre-operation	10.63 ± 10.95	13.06 ± 11.91	0.444
Post-operation	− 0.7 ± 1.02	− 0.12 ± 1.07	0.859
Final follow-up	− 0.06 ± 1.00	− 0.10 ± 0.95	0.905
Reduction loss (°)	0.01 ± 0.37	0.03 ± 0.43	0.861

## Discussion

The outcome of this study shows that the isolated bone grafting for the treatment of PTPCF could achieve a satisfactory range of motion of knee joint (a mean extension of 0.65° ± 1.72° and a mean flexion of 124.78° ± 7.46°) and HSS scores (95.57 ± 1.9) and Rasmussen score (28.22 ± 0.85). Furthermore, the fixation with isolated bone grafting for the treatment of PTPCF could get a relatively stable fixation without significantly loss of reduction compare to fixation with cannulated screws with similar posterior inclination angle reduction loss and lateral inclination angle reduction loss.

ACL injuries are often accompanied by tibial plateau fractures [9, 13, 14]. A meta-analysis of knee MRIs in 1047 cases of ACL injuries revealed that early knee MRIs can detect bone contusion signal changes in up to 78% of patients [15]. The presence of tibial plateau bone contusion or fractures on MRI, and even impaction fractures of the tibial plateau and femoral lateral condyle, usually signifies severe knee joint trauma caused by violent force leading to knee joint subluxation [2]. Tibial plateau bone contusions or impaction fractures, often combined with femoral lateral condyle injuries, are believed to occur during the process of ACL injury, which involves knee joint flexion, external rotation, and valgus force. During the reduction process after knee joint subluxation, the impact between the lateral femoral condyle and the tibial plateau's posterolateral aspect is responsible for this injury. A typical tibial plateau impaction fracture manifests as a coronal plane defect in the posterolateral tibial plateau, hence it has been termed the "bitten apple" fracture [16, 17].

Bernholt et al. categorized tibial plateau compression fractures into three major classes based on the MRI presentation of the lateral tibial plateau: posterior cortical fractures of the tibial plateau that do not involve the articular surface, posterior cortical fractures of the tibial plateau that affect the articular surface, and posterior split fractures of the tibial plateau [18]. Building upon this classification, Menzdorf and colleagues employed the posterior horn of the lateral meniscus as a reference point to aid in the reclassification of surgical guidelines, though the substantial individual variability of meniscus morphology led to a reduction in classification reliability [17].

However, the two fracture classifications did not encompass the tibial plateau compression fractures included in this study. Tibial plateau compression fractures, often treated with arthroscopically assisted reduction and cannulated screw fixation involving the use of a metal tamp during the reduction process, exhibit various deficiencies, such as articular surface fracture fragment fragmentation, uneven articular surfaces, inadequate angular reduction, and fracture fragment rotation. To facilitate the use of metal tamp for fracture reduction, the authors categorized compression fractures of the lateral tibial plateau into three types based on the primary direction of articular surface inclination: posterior inclination, lateral inclination, and horizontal compression types [10]. In this study, the main focus was on posterior inclination and lateral inclination types. In contrast to tibial plateau impaction fractures accompanied by ACL injury, which often display the "kissing sign" on knee MRI [19], this sign is less frequent in the MRIs of knee joints with tibial plateau compression fractures associated with ACL injuries. The mechanism behind this difference might be that the lateral tibial plateau compression during the violent process of flexion and external rotation increases the posterior inclination angle or external rotation angle. This increased stress on the ACL eventually leads to ACL injury, even without knee joint subluxation. Tibial plateau articular subsidence and increased posterior inclination angle are closely related to the posterolateral instability of the knee joint and are often associated with a positive pivot-shift test of grade 2 or higher in knee joints [20]. A posterior inclination angle of 17 degrees or more is a significant factor in post-ACL reconstruction failure [21]. Thus, the treatment of tibial plateau compression fractures has attracted considerable attention.

**Fig. 3:**
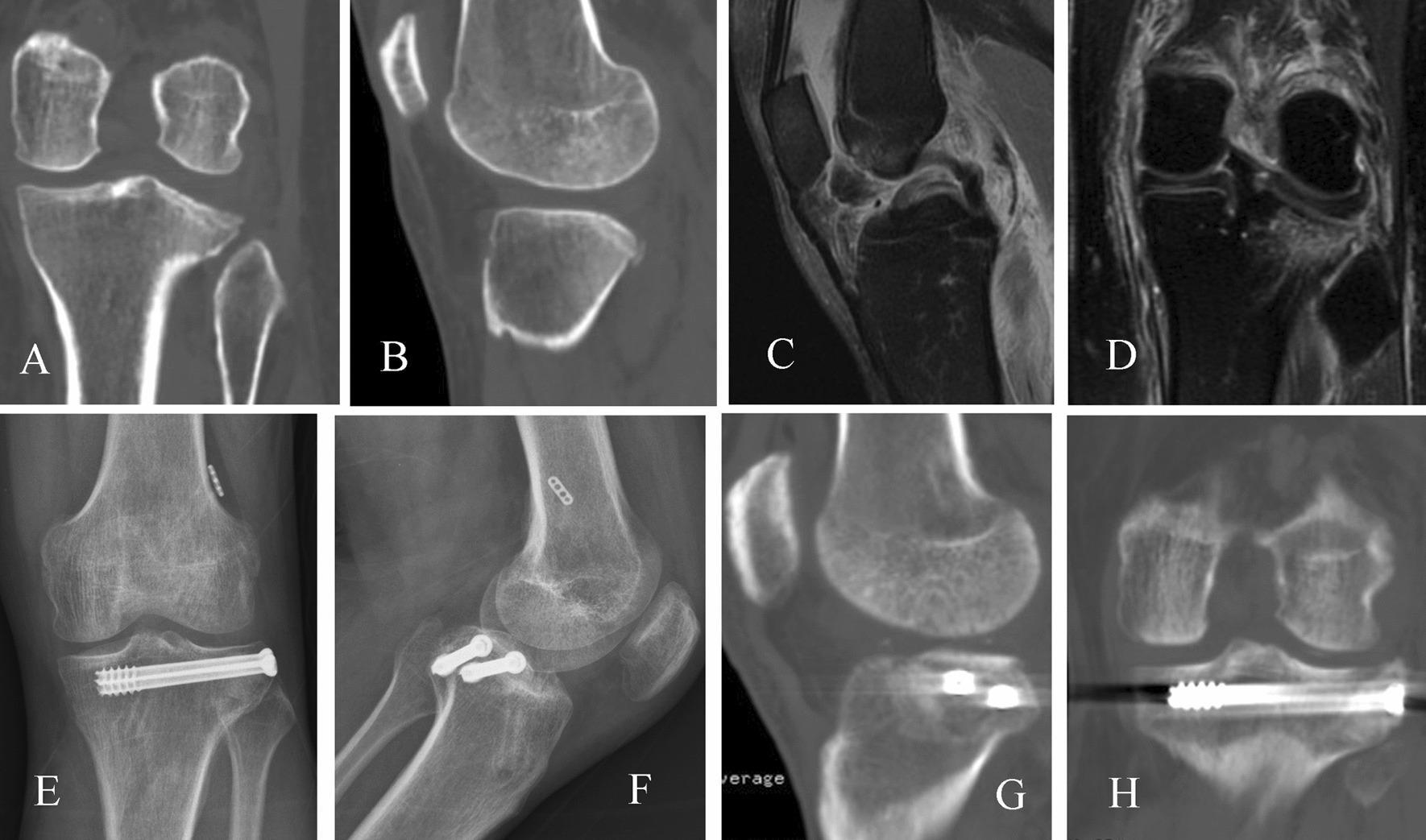
A 55-year-old male patient was admitted due to left knee pain following a fall from an electric bicycle. 3DCT scans of the left knee upon admission revealed a compressive fracture of the posterolateral tibial plateau. The coronal view demonstrated a significantly increased lateral inclination of the lateral tibial plateau (**A**), and the sagittal view displayed an increased posterior inclination (**B**). The sagittal view of the MRI revealed an acute ACL tear (**C**), while the coronal view of the MRI also indicated a lateral tibial plateau compression fracture (**D**). The patient underwent a one-stage procedure involving ACL reconstruction and arthroscopically assisted reduction and fixation of the PTPCF using two cannulated screws without bone grafting. Fourteen months post-surgery, the fracture fragments exhibited successful union with no significant reduction loss (**E**–**H**)

**Fig. 4:**
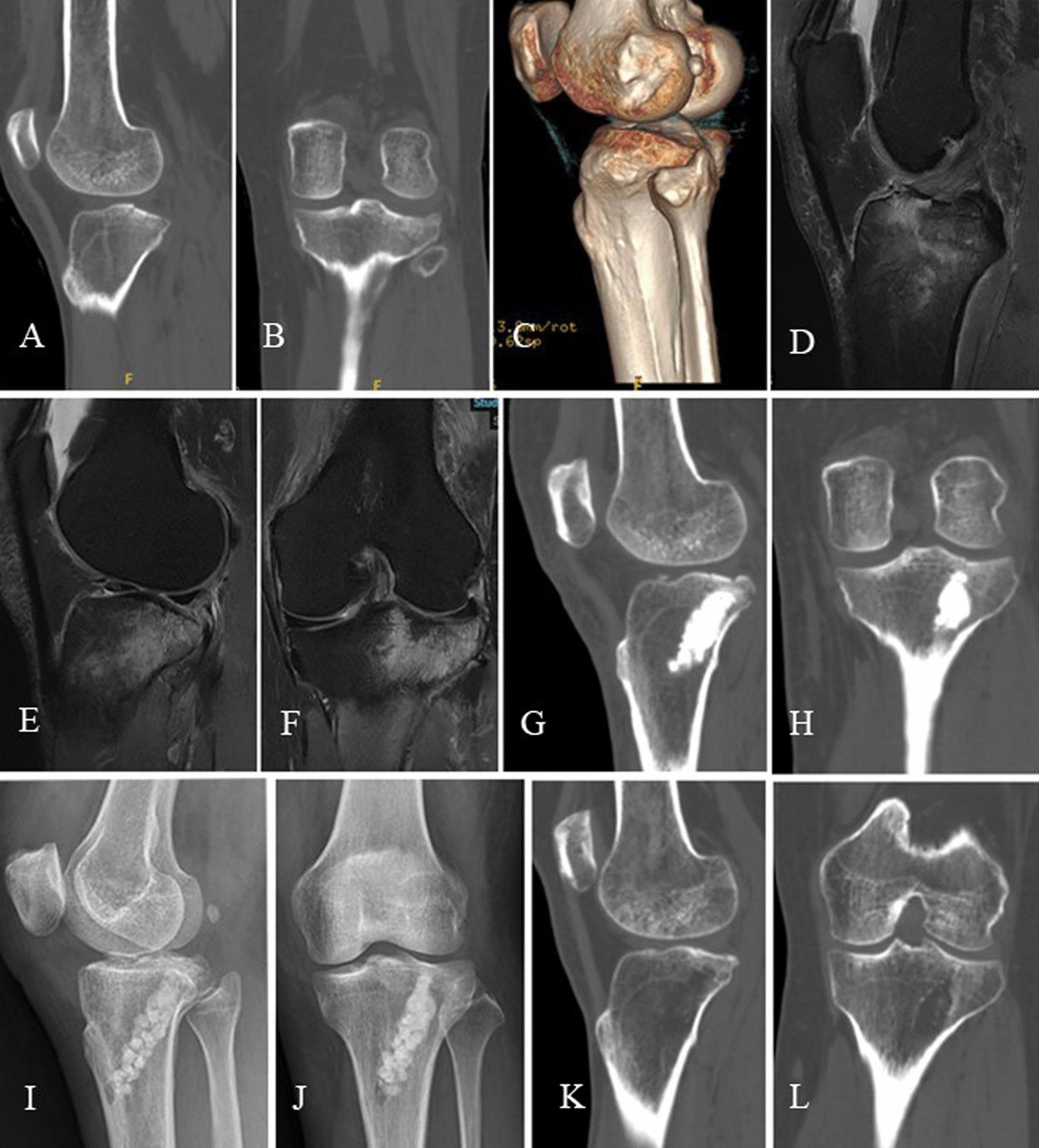
A 41-year-old female patient admitted due to limited knee mobility and pain caused by a car accident-related injury to the left knee. 3DCT scans of the knee upon admission revealed a compressive fracture of the posterolateral tibial plateau. The sagittal view displayed significant increased relative posterior inclination of the lateral plateau (**A**), while the coronal view showed partial compression with less lateral inclination (**B**). Three-dimensional reconstruction illustrated a significant increase in the lateral plateau's relative posterior slope (**C**). Subsequent knee joint MRI revealed an avulsion fracture at the attachment of the ACL (**D**), posterolateral plateau collapse with pronounced lateral plateau edema, and no signs of femoral condyle edema (**E**, **F**). Arthroscopically assisted fixation of the anterior cruciate ligament avulsion fracture was performed, and in a single-stage procedure, the lateral plateau fracture was restored and securely fixed using calcium sulfate artificial bone grafting, resulting in an anatomical reduction of joint surface (**G**–**J**). Two years later, a follow-up 3DCT scan showed excellent fracture healing with some bone resorption within the tunnels (**K, L**)

The treatment of tibial plateau compression fractures varies. As arthroscopic technology continues to advance, many orthopedists have adopted arthroscopic reduction and cannulated screw fixation, which has shown good results [11, 12]. However, excessive cannulated screw fixation may interfere with the tibial tunnel for the ACL, affect grafts, risk peroneal nerve damage during the lateral implantation of the cannulated screw fixation, and require a second removal of the cannulated screw fixation [22, 23]. In this retrospective study, we compared the clinical and radiographic results of arthroscopic reduction and cannulated screw fixation and isolated bone grafting for tibial plateau compression fractures. It was found that simply filling and compacting bone under the tibial plateau fracture fragment can achieve a good reduction support effect. There were no significant differences in postoperative knee joint function or reduction loss between two groups. The subchondral bone underlying the fracture fragment in the tibial plateau is characterized by its trabecular composition, imposing rigorous requirements on the strategic placement of cannulated screws. Therefore, it is imperative to position the screws directly beneath the fracture fragment of the articular surface to attain an efficacious support effect (Fig. 3). In this study, increasing the trabecular bone density under the fracture fragment by performing allograft bone or calcium sulfate artificial bone grafting and compaction under the articular surface fracture fragment results in good reduction support, as shown in Fig. 4. Notably, neither the application of one or two cannulated screws nor isolated bone grafting can achieve the level of robust fixation compared with plate fixation [24, 25]. During knee joint movement, the axial load on the lateral tibial plateau is minimal when in the extended position. However, during knee flexion, due to the roll-back effect of the lateral condyle of the femur, the lateral plateau gradually begins to bear axial stress [26, 27]. Therefore, regardless of whether cannulated screw internal fixation is used, early weight-bearing knee flexion exercises should be avoided during postoperative rehabilitation.

In this study, some patients were managed using calcium sulfate artificial bone for grafting. Calcium sulfate artificial bone has good biocompatibility and degradability, while also inducing bone formation and having excellent mechanical properties. Although some degradation and absorption were observed during postoperative follow-up, all fractures achieved good healing, with no reduction loss (Fig. 4). Other patients were under went allograft bone grafting for the fixation of PTPCF. While the mechanical performance of allograft bone is lower than that of calcium sulfate artificial bone, its absorption rate is lower, approximately 10.78% [28]. During the follow-up, the fracture healed well with no significant loss of reduction. In comparison to cannulated screw fixation group, bone grafting fixation have several advantages, including avoiding the use of additional metal implants, reducing the risk of peroneal nerve injury during the fixation process, and eliminating the need for secondary removal surgery.

### Limitations

Limitations of this study include its retrospective study design, a relatively small sample size, a short follow-up period, and inherent selection bias regarding the use of metal cannulated screw fixation during the surgical process. Additionally, the study did not include patients with tibial plateau compression fractures who had conservative treatment for the tibia plateau without ACL injuries, resulting in a lack of a control group. Furthermore, the study did not provide a more reasonable classification of tibial plateau compression fractures, and it is essential to determine whether simply bone grafting defects under the tibial plateau suffice for achieving ideal reduction and fixation, depending on the type of tibial plateau fracture.

## Conclusions

The use of isolated bone grafting for fixation of PTPCF with accompanying ACL injuries demonstrated no substantial disparities in postoperative knee joint range of motion, Rasmussen score, HSS knee joint function score, or VAS score when contrasted with cannulated screw fixation. In cases of simple PTPCF without lateral wall disruption, filling and compacting the bone defect underneath the tibial plateau fracture fragment can yield satisfactory fixation results, obviating the necessity for supplementary cannulate screw fixation.

## Data Availability

Not applicable.

## Abbreviations

PTPCF: Posterolateral tibial plateau compression fractures
ACL: Anterior cruciate ligament
MRI: Magnetic resonance imaging
3DCT: Three-dimensional computed tomographs
VAS: Visual analogue scale
BMI: Body mass index
HSS: Hospital for Special Surgery
